# Supplementary material for: The Effects of Thermocycling on the Physical Properties and Biocompatibilities of Various CAD/CAM Restorative Materials
Source: Pharmaceutics. 2023 Aug 10;15(8):2122. doi: 10.3390/pharmaceutics15082122 (PMC10459511; doi:10.3390/pharmaceutics15082122)
Supplement: Supplementary file 1 [file pharmaceutics-15-02122-s001.zip › Supplementary Table 1 (revised).pdf]

Supplementary Table 1. Mean and SD values of nanoindentation hardnesses (GPa).

Group	Mean ± SD (GPa)					
	Control	1st aged	***P***	2nd aged	***P*** †	***P*** ‡
M	7.85±1.22[b]	5.15±0.81[b]	<.001*	4.72±1.02[b]	<.001*	.096
C	7.79±1.16[b]	6.10±0.76[b]	<.001*	5.81±0.78[b]	<.001*	.170
E	7.05±0.92[b]	5.09±2.79[b]	<.001*	4.17±3.02[b]	<.001*	.252
S	0.97±0.25[a]	0.80±0.24[a]	.011*	0.77±0.30[a]	.009*	.697
Z	19.84±3.09[c]	19.79±2.87[c]	.954	19.35±3.63[c]	.599	.625

M: IPS e.max CAD, C: Celtra Duo, E: Vita Enamic, S: Cerasmart, Z: Lava™ Plus Zirconia.
Different superscripted letters in each vertical column indicate significant differences (P <0.05).
P value were calculated by result of independent samples t-test between control groups and first aged groups.
P† value were calculated by result of independent samples t-test between control groups and second aged groups.
P‡ value were calculated by result of independent samples t-test between first aged groups and second aged groups.
