# Supplementary material for: The Effects of Thermocycling on the Physical Properties and Biocompatibilities of Various CAD/CAM Restorative Materials
Source: Pharmaceutics. 2023 Aug 10;15(8):2122. doi: 10.3390/pharmaceutics15082122 (PMC10459511; doi:10.3390/pharmaceutics15082122)
Supplement: Supplementary file 1 [file pharmaceutics-15-02122-s001.zip › Supplementary Table 2 (revised).pdf]

**Supplementary Table 2.** Means and standard deviations of the Young's moduli (GPa).

| Group | Mean ± SD (GPa) | | | | | |
|---|---|---|---|---|---|---|
| | Control | 1st aged | ***P*** | 2nd aged | ***P*** † | ***P*** ‡ |
| M | 101.43±11.35 [c] | 80.48±8.56 [c] | <.001* | 74.16±10.12 [c] | <.001* | .016* |
| C | 102.22±7.57 [c] | 73.16±7.04 [c] | <.001* | 72.45±5.76 [c] | <.001* | .680 |
| E | 58.91±7.05 [b] | 45.78±18.38 [b] | <.001* | 39.22±18.98 [b] | <.001* | .203 |
| S | 12.69±1.82 [a] | 11.60±1.93 [a] | .038* | 11.29±2.13 [a] | .013* | .579 |
| Z | 252.70±38.57 [d] | 238.67±25.74 [d] | .122 | 224.39±29.99 [d] | .004* | .066 |

M: IPS e.max CAD, C: Celtra Duo, E: Vita Enamic, S: Cerasmart, Z: LavaTM Plus Zirconia.
Different superscripted letters of each vertical column indicate significant differences (P <0.05).
*P* value were calculated by result of independent samples t-test between control groups and first aged groups.
*P*† value were calculated by result of independent samples t-test between control groups and second aged groups.
*P*‡ value were calculated by result of independent samples t-test between first aged groups and second aged groups.
