# Supplementary material for: The Effects of Thermocycling on the Physical Properties and Biocompatibilities of Various CAD/CAM Restorative Materials
Source: Pharmaceutics. 2023 Aug 10;15(8):2122. doi: 10.3390/pharmaceutics15082122 (PMC10459511; doi:10.3390/pharmaceutics15082122)
Supplement: Supplementary file 1 [file pharmaceutics-15-02122-s001.zip › Supplementary Table 3 (revised).pdf]

Supplementary Table 3. Mean ± SD values and statistical analyses of surface roughnesses (R_a and R_q).

Surface roughness	Group	Mean ± SD (nm)					
		Control	1st aged	*P*	2nd Aged	*P*†	*P* ‡
	M	4.82 ± 0.71 [b]	5.00 ± 0.82 [b]	.400	5.33±1.21 [b]	.067	.249
	C	4.44±0.51 [b]	6.26±2.53 [b]	<.001*	6.82±0.86 [b]	<.001*	.280
R_a	E	6.68±1.09 [c]	8.55±2.67 [c]	<.001*	11.41±4.67 [c]	<.001*	<.009*
	S	13.08±1.37 [d]	13.55±1.15 [d]	.173	15.00±1.79 [d]	<.001*	<.009*
	Z	2.82±0.48 [a]	2.29±0.28 [a]	.351	3.21±0.41 [a]	.002*	.004*
	M	6.38±0.98 [b]	6.76 ±1.28 [a,b]	.232	7.30±1.86 [a,b]	<.026*	.213
	C	6.44±0.85 [b]	8.60±3.34 [b]	.002*	9.83±0.92 [b]	<.001*	.070
R_q	E	10.02±1.70 [c]	15.73±7.67 [c]	<.001*	21.38±8.57 [c]	<.001*	.014*
	S	17.74±1.80 [d]	19.12±1.61 [d]	.005*	21.90±2.98 [c]	<.001*	.004*
	Z	3.81±0.59 [a]	4.00±0.35 [a]	.157	4.32±0.56 [a]	.002*	.014*

M: IPS e.max CAD, C: Celtra Duo, E: Vita Enamic, S: Cerasmart, Z: Lava Plus Zirconia
Different superscripted letters of each vertical column indicate significant differences ($P<0.05$).
P value were calculated by result of independent samples t-test between control groups and first aged groups.
P† value were calculated by result of independent samples t-test between control groups and second aged groups.
P‡ value were calculated by result of independent samples t-test between first aged groups and second aged groups.
