# Supplementary material for: The Effects of Thermocycling on the Physical Properties and Biocompatibilities of Various CAD/CAM Restorative Materials
Source: Pharmaceutics. 2023 Aug 10;15(8):2122. doi: 10.3390/pharmaceutics15082122 (PMC10459511; doi:10.3390/pharmaceutics15082122)
Supplement: Supplementary file 1 [file pharmaceutics-15-02122-s001.zip › Supplementary Table 4 (reivised).pdf]

Supplementary Table 4. Means and standard deviations of the contact angles (degrees).

Group	Mean ± SD (degrees)					
	Control	1st aged	***P***	2nd aged	***P*** †	***P*** ‡
M	54.04±16.61[a]	38.58±11.17 [a]	<.001*	37.43±10.96 [b]	<.001*	.663
C	75.31±5.16 [b]	69.84±5.12 [c]	<.001*	28.11±12.43 [a]	<.001*	<.001*
E	56.41±12.96 [a]	53.73±12.56 [b]	.375	48.49±10.58 [c]	.006*	.060
S	61.79±7.79 [a b]	59.44±7.50 [b,c]	.198	55.97±8.55 [c]	.004*	.071
Z	96.08±41.19 [c]	95.09±48.30 [d]	.925	76.94±17.19 [d]	.012*	.037*

M: IPS e.max CAD, C: Celtra Duo, E: Vita Enamic, S: Cerasmart, Z: Lava Plus Zirconia

Different superscripted letters of each vertical column indicate significant differences ($P<0.05$).

P value were calculated by result of independent samples t-test between control groups and first aged groups.

$P^{\dagger}$ value were calculated by result of independent samples t-test between control groups and second aged groups.

$P^{\ddagger}$ value were calculated by result of independent samples t-test between first aged groups and second aged groups.
